# Prevalence of Antimicrobial Resistance in Select Bacteria From Retail Seafood—United States, 2019

**DOI:** 10.3389/fmicb.2022.928509

**Published:** 2022-06-23

**Authors:** Heather Tate, Sherry Ayers, Epiphanie Nyirabahizi, Cong Li, Stacey Borenstein, Shenia Young, Crystal Rice-Trujillo, Sanchez Saint Fleurant, Sonya Bodeis-Jones, Xunde Li, Melissa Tobin-D’Angelo, Victoriya Volkova, Rachel Hardy, Lisa Mingle, Nkuchia M. M’ikanatha, Laura Ruesch, Chris A. Whitehouse, Gregory H. Tyson, Errol Strain, Patrick F. McDermott

**Affiliations:** ^1^Center for Veterinary Medicine, U.S. Food and Drug Administration, Laurel, MD, United States; ^2^School of Veterinary Medicine, University of California, Davis, Davis, CA, United States; ^3^Acute Disease Epidemiology Section, Georgia Department of Public Health, Atlanta, GA, United States; ^4^Department of Diagnostic Medicine/Pathobiology, College of Veterinary Medicine, Kansas State University, Manhattan, KS, United States; ^5^Missouri State Public Health Laboratory, Jefferson City, MO, United States; ^6^Wadsworth Center Division of Infectious Diseases, New York State Department of Health, Albany, NY, United States; ^7^Division of Infectious Disease Epidemiology, Pennsylvania Department of Health, Harrisburg, PA, United States; ^8^Animal Disease Research and Diagnostic Laboratory, Veterinary and Biomedical Sciences Department, South Dakota State University, Brookings, SD, United States

**Keywords:** antimicrobial resistance, seafood, retail food, United States, National Antimicrobial Resistance Monitoring System

## Abstract

In 2019, the United States National Antimicrobial Resistance Monitoring System (NARMS) surveyed raw salmon, shrimp, and tilapia from retail grocery outlets in eight states to assess the prevalence of bacterial contamination and antimicrobial resistance (AMR) in the isolates. Prevalence of the targeted bacterial genera ranged among the commodities: *Salmonella* (0%–0.4%), *Aeromonas* (19%–26%), *Vibrio* (7%–43%), *Pseudomonas aeruginosa* (0.8%–2.3%), *Staphylococcus* (23%–30%), and *Enterococcus* (39%–66%). Shrimp had the highest odds (OR: 2.8, CI: 2.0–3.9) of being contaminated with at least one species of these bacteria, as were seafood sourced from Asia vs. North America (OR: 2.7; CI: 1.8–4.7) and Latin America and the Caribbean vs. North America (OR: 1.6; CI: 1.1–2.3) and seafood sold at the counter vs. sold frozen (OR: 2.1; CI: 1.6–2.9). Isolates exhibited pan-susceptibility (*Salmonella* and *P. aeruginosa*) or low prevalence of resistance (<10%) to most antimicrobials tested, with few exceptions. Seafood marketed as farm-raised had lower odds of contamination with antimicrobial resistant bacteria compared to wild-caught seafood (OR: 0.4, CI: 0.2–0.7). Antimicrobial resistance genes (ARGs) were detected for various classes of medically important antimicrobials. Clinically relevant ARGs included carbapenemases (*bla*_IMI-2_, *bla*_NDM-1_) and extended spectrum β-lactamases (ESBLs; *bla*_CTX-M-55_). This population-scale study of AMR in seafood sold in the United States provided the basis for NARMS seafood monitoring, which began in 2020.

## Highlights

- The prevalence of seafood contamination with resistant bacteria was low.
- Carbapenemases (*bla*_IMI-2_, *bla*_NDM-1_) and ESBLs (*bla*_CTX-M-55_) were found in imported seafood.
- This work provides the basis for national tracking of seafood-borne AMR in the United States.

## Introduction

An increasing number of Americans are consuming seafood. In 2019, the United States *per capita* annual seafood consumption reached 19.2 pounds (National Marine Fisheries Service, 2021), an increase of 1.5 pounds compared to *per capita* consumption in 2010. While lower than beef (57.9 pounds *per capita* in the United States in 2019) and chicken (95.1 pounds *per capita* in the United States in 2019; U.S. Department of Agriculture Economic Research Service, 2021), consumption of seafood is expected to increase. This trend is similar to that worldwide, reflecting rising incomes, transitions in dietary preferences and nutrition guidelines, and associated increases in production (Schar et al., 2020; U.S. Food and Drug Administration, 2020a). As of 2019, shrimp is the most consumed seafood product in the United States (4.7 pounds *per capita*), followed by salmon (3.1 pounds *per capita*), canned tuna (2.2 pounds *per capita*), Alaska pollock (0.996 pounds *per capita*), and tilapia (0.98 pounds *per capita*; National Fisheries Institute, 2022).

Aquaculture has grown over the decades to meet the increased consumer demand and improve sustainability of the food supply. Aquaculture now accounts for almost 50% of total seafood production (Food and Agriculture Organization of the United Nations, 2020). Seafood grown in aquaculture farms (as opposed to wild-caught seafood) are more likely to be exposed to antimicrobials, which are fed to fish and shellfish to combat disease resulting from intensive husbandry practices. There are only three antimicrobial drug classes (tetracyclines, phenicols, and potentiated sulfonamides) approved for the treatment and control of bacterial disease in aquaculture raised in the United States (U.S. Food and Drug Administration, 2020b). However, 65%–90% of seafood marketed in the United States is imported (Gephart et al., 2019; National Marine Fisheries Service, 2021) and may be exposed to additional drug classes (Schar et al., 2020), and many of these drugs are also used in human medicine (Smith, 2008). The use of antimicrobial drugs in aquaculture can precipitate the emergence of AMR in zoonotic pathogens that can then be directly transmitted to humans through consumption of or contact with contaminated product. Seafood can be a source of bacterial illness for humans in the United States. In 2010, the Centers for Disease Control and Prevention reported 143 seafood-associated outbreaks caused by a bacterial agent occurred in the United States during 1973 to 2006, with *Vibrio parahaemolyticus* being the most commonly reported agent (Iwamoto et al., 2010). AMR among seafood-borne bacteria could potentially decrease the effectiveness of antimicrobial treatments in humans. Also concerning, is the possibility of indirect transmission, or the enrichment and horizontal transfer of ARGs from bacteria in aquatic environments to related human pathogens.

A number of studies have shown that seafood are carriers of antimicrobial resistant bacteria (Zhao et al., 2003; Han et al., 2007; Khan et al., 2009; Tran et al., 2011; Wang et al., 2011; Nawaz et al., 2012; Ryu et al., 2012; Shaw et al., 2014; Boss et al., 2016; Elbashir et al., 2018; Jans et al., 2018). Recognizing that seafood could be a potential point of origin for emergence and enrichment of antimicrobial resistant bacteria in the United States, in 2020 the NARMS began national monitoring of resistant bacteria in retail seafood. To establish the optimal criteria for routine systematic seafood surveillance and monitoring and to estimate the resistant bacteria status of retail seafood sold in the United States, in 2019 NARMS conducted a year-long pilot study collecting retail raw shrimp, tilapia, and salmon from grocery outlets in eight states across the country. For the pilot study, we evaluated, in retail seafood, bacteria of public health importance (*Salmonella*, *Pseudomonas aeruginosa*, *Vibrio* spp.) and both spoilage-related and naturally occurring aquatic bacteria (*Staphylococcus* spp., *Enterococcus* spp., *Aeromonas* spp.) that were known to have sufficiently high prevalence in fish and shellfish (Tuševljak et al., 2012; Boss et al., 2016; Elbashir et al., 2018). We also looked for carbapenem-resistant organisms (CRO), using selective enrichment methods. The prevalence of the targeted bacterial genera, their AMR patterns, and ARG findings are presented herein. We also tested the statistical significance of associations between epidemiologic variables and both bacterial species of major and minor public health importance and the occurrence of antimicrobial resistant bacteria.

## Materials and Methods

### Sampling

Between January and December of 2019, samples of salmon (*n* = 710) and shrimp (*n* = 710) were purchased from supermarkets in participating NARMS sites: California, Georgia, Kansas, Missouri, New York, Pennsylvania, South Carolina, and South Dakota. Seafood was collected as fresh, frozen, or previously frozen raw samples. Tilapia (*n* = 214) also was collected from supermarkets in the above states (except for Pennsylvania) between September and December of 2019. A sample was defined as the unit that was purchased, and may have varied by purchase. For example sometimes a one pound bag of peeled frozen shrimp may have been purchased and other times a quarter pound of individual head-on-tail-on shrimp were purchased. Country of origin, raising claims (antibiotic use vs. organic and farm-raised vs. wild-caught), meat cut, and salmon variety were recorded for all samples, where applicable. Participating laboratories performed sample collection and preparation, and bacterial isolation.

### Sample Preparation

A 25 gram portion of each seafood sample was aliquoted with 225 ml of Alkaline Peptone Water (APW, *Vibrio*; Thermo-Scientific, Waltham, MA), 225 ml lactose broth (*Salmonella*), or 225 ml Buffered Peptone Water (BPW; BD Difco^™^, Detroit, MI; *Enterococcus*, *Aeromonas*, *P. aeruginosa*, *Staphylococcus*, CRO) in a sterile stomacher bag and stomached or blended for 2 min, followed by incubation at 35°C for 24 h. Unless otherwise noted, the enriched mixture (1 μl) was streaked onto agar plates described below.

### Bacterial Isolation and Identification

All samples were tested for *Vibrio*, *P. aeruginosa*, *Enterococcus*, *Staphylococcus*, and *Aeromonas*. However, only 506 salmon, 498 shrimp and 205 tilapia samples were tested for *Salmonella* as testing was stopped midway through the pilot due to low recovery. All samples collected were evaluated for the presence of CROs except for tilapia because testing was stopped midway due to the detection of intrinsic resistance mechanisms in the majority of sequenced isolates. For *Vibrio*, APW enrichments were streaked onto Thiosulfate-Citrate-Bile-Sucrose agar (BD Difco^™^) and incubated at 35°C for 18–24 h. Presumptive *Vibrio* characteristically appeared as green or yellow colonies. If more than one color appeared, one of each colored colony was selected. For *Salmonella*, 0.1 ml of lactose broth enrichment was transferred to 10 ml Rappaport-Vassiliadis (RVR10) medium (BD Difco^™^) and incubated at 42°C for 24 h. Each RVR10 culture was streaked to one XLT-4 (Thermo-Scientific, Remel^™^, Lenexa, KS) and one HE agar (Thermo-Scientific, Remel^™^) and incubated at 35°C for 24 h. One typical colony was selected per agar for further testing. For *P. aeruginosa*, BPW enrichments were streaked to either *Pseudomonas* Isolation Agar (PIA; BD Difco^™^) or MacConkey agar (MAC; Thermo-Scientific) and plates were incubated at 35°C for 18–48 h. *Pseudomonas* spp. appeared as colorless colonies on MAC and presumptive *P. aeruginosa* characteristically appeared as green/blue–green colonies on PIA. One colony was selected per sample. For *Enterococcus*, BPW enrichments were streaked to Enterococcosel agar (BD BBL^™^, Franklin Lakes, NJ) or Mannitol Salt Agar (MSA; BD BBL^™^) and incubated for 48 h at 35°C. One colony was selected from each sample. *Staphylococcus* were also isolated from MSA. After streaking with BPW enrichments, plates were incubated for 24 h at 35°C. One colony was selected per sample. For isolation of *Aeromonas* Cefsulodin-Irgasan-Novobiocin (CIN) agar (Thermo-Scientific, Remel^™^) was incubated for 24–48 h at 25°C. Presumptive *Aeromonas* colonies had a pale colonies with rose red centers. One colony was selected per sample. Isolation of CRO bacteria was performed by streaking BPW enrichments onto mSuperCarba CHROMagar^™^ (CHROMagar^™^, France) and incubating at 35°C–37°C for 18–24 h. One colony of each color (up to five from each sample) was picked. According to the package insert, the typical appearance of carbapenemase producing microorganisms is dark pink to red (*E.coli*), metallic blue (Coliforms), translucent, +/− natural pigmentation cream to green (*Pseudomonas*), Cream (*Acinetobacter*), colorless, natural pigmentation (other Gram-negative organisms). For all targeted bacteria, presumptive isolates were picked from their respective selective media, streaked to blood agar plates (BAP; Thermo-Scientific^™^) and incubated at 35°C for 24 h. If growth was pure, colonies were swabbed to *Brucella* broth (BD Difco^™^) with 15% glycerol mixture, frozen at −60°C to −80°C, and shipped to the United States Food and Drug Administration (FDA) Center for Veterinary Medicine (CVM) for additional analyses.

At FDA, all bacterial isolates received were confirmed using the VITEK^®^ 2 Compact (*n* = 6,491; bioMérieux, France), except for isolates grown on mSuperCarba CHROMagar^™^. Only a subset of presumptive mSuperCarba CHROMagar^™^ isolates (*N* = 952) were confirmed on the VITEK^®^ 2 Compact.

### Antimicrobial Susceptibility Testing

*Salmonella* spp., *P. aeruginosa*, *Vibrio* spp., *Staphylococcus* spp., *Enterococcus*, and *Aeromonas* spp. isolates were tested for susceptibility to antimicrobials approved for use in aquaculture in the United States as well as other antimicrobial classes that may be used in other countries. Testing was performed at FDA CVM *via* the broth microdilution assay (Sensititre^™^ System, Thermo Fisher Scientific^™^) using methods recommended by the Clinical and Laboratory Standards Institute (CLSI; Clinical and Laboratory Standards Institute, 2016, 2019). Gram-negative bacteria were tested on either Gram-negative antimicrobial panel (Sensititre^™^ panel CMV4AGNF or CMV5AGNF, Thermo Fisher Scientific^™^). All 3 *Salmonella* and 20 *P. aeruginosa* underwent antimicrobial susceptibility testing (AST). Due to the large number of isolates recovered, a random subset of 179 *Aeromonas* isolates underwent AST. To examine associations between sample-based variables (e.g., geographic region of origin) and isolate-based variables (e.g., AMR) as described below, only one *Vibrio* isolate per positive sample was selected for AST. Of the selected isolates, all isolates from the following species were subjected to AST: *V. parahaemolyticus* (*n* = 91), *V. cholerae* (*n* = 30), and *V. vulnificus* (*n* = 1); and we tested a random subset of isolates of each species: *V. metschnikovii* (*n* = 53), *V. fluvialis* (*n* = 27), *V. alginolyticus* (*n* = 5) and mixed populations (*n* = 3). The antimicrobial classes tested on both panels were: aminoglycosides (gentamicin), β-lactam combination agents (amoxicillin-clavulanic acid), carbapenems (meropenem), cephems (cefoxitin, ceftriaxone), folate pathway inhibitors (sulfisoxazole and trimethoprim-sulfamethoxazole), macrolides (azithromycin), penicillins (ampicillin), phenicols (chloramphenicol), quinolones (ciprofloxacin, nalidixic acid), and tetracyclines (tetracycline). Isolates tested on the Sensititre^™^ CMV5AGNF panel were also tested against lipopeptides (colistin).

We tested 385 *Enterococcus* and 210 *Staphylococcus* isolates on the NARMS Gram-positive antimicrobial panel (Sensititre^™^ panel CMV4AGP, Thermo Fisher Scientific) that included the following antimicrobial classes and drugs: aminoglycosides (gentamicin, streptomycin), glycopeptides (vancomycin), glycylcyclines (tigecycline), lipopeptides (daptomycin), macrolides (erythromycin), nitrofurans (nitrofurantoin), orthosomycins (avilamycin), oxazolidones (linezolid), penicillins (ampicillin), phenicols (chloramphenicol), streptogramins (quinupristin-dalfopristin), quinolones (ciprofloxacin), and tetracyclines (tetracycline).

Interpretation of minimum inhibitory concentration (MIC) values was based on the CLSI clinical breakpoints for human infection treatment, when available (Clinical and Laboratory Standards Institute, 2016, 2019). Otherwise NARMS provisional cutoffs were used for azithromycin against *Salmonella* (interpreted as resistant if MIC ≥ 32 μg/ml) and tigecycline against *Enterococcus* (interpreted as resistant if MIC > 0.25 μg/ml; U.S. Food and Drug Administration, 2019). Because the isolates recovered from mSuperCarba CHROMagar^™^ did not undergo confirmatory phenotypic susceptibility testing for carbapenem antibiotics, those isolates are referred to as “presumptive CRO” from this point forward.

### Identification of ARGs

A subset of presumptive CRO was randomly selected for whole genome sequencing (WGS) and all *Salmonella* were sequenced. Of the other targeted genera (*Enterococcus*, *P. aeruginosa*, *Staphylococcus* spp., *Vibrio* spp., and *Aeromonas* spp.), we performed WGS only on a subset of the isolates with resistance to at least one antimicrobial. Altogether, 370 isolates were sequenced including 3 *Salmonella*, 16 *Aeromonas*, 2 *Enterococcus*, 6 *P. aeruginosa*, 11 *Staphylococcus*, 44 *Vibrio* and 288 presumptive CRO. Isolates were sequenced on Illumina MiSeq^™^ using v3 reagent kits (Illumina Inc., San Diego, CA, United States) with 2 × 300 bp paired-end reads. The libraries were prepared with Nextera XT kit by Illumina, and raw sequences were assembled *de novo* using CLC Genomic Workbench (version 10.0). ARGs were identified in the assembled genomes with AMRFinder Plus 3.8 software.^1^ All isolate identifiers are listed in Supplementary Table S1, and can be found under NCBI Bioproject PRJNA800017. Speciation was done using the MLST software^2^ which incorporates components of the PubMLST database^3^ (Jolley and Maiden, 2010). WGS speciation was crosschecked with the VITEK^®^ identification and when they differed, the WGS speciation was chosen for all analysis. If bacterial genera could not be resolved through *in silico* methods, VITEK^®^ identification was used.

### Statistical Analysis

The percent of samples that tested positive was calculated by dividing the number of samples that yielded the bacterial genera or species by the total number of samples of the seafood commodity collected.

The percent phenotypically resistant was calculated by dividing the number of isolates with the antimicrobial MIC at or above the CLSI clinical breakpoint for human infection treatment or the NARMS cutoff interpretive criterion by the total number of isolates of that bacterial species tested against that antimicrobial. Percent resistance was only calculated for antimicrobials with CLSI breakpoints or NARMS interpretive criteria. Isolates resistant to at least three classes of antimicrobials were considered multidrug resistant.

Two sets of multivariable logistic regression models were used (PROC LOGISTIC, SAS version 9.4 (SAS, Cary, NC)) to evaluate the association for a sample between epidemiologic risk factors (Table 1) and the following outcomes: (1) being culture positive for at least one of the targeted genera (*Aeromonas*, *Salmonella*, *Staphylococcus*, *Enterococcus*, *P. aeruginosa*, or *Vibrio*), or (2) contamination of a sample with an antimicrobial resistant isolate of the target bacterial genera (i.e., excluding presumptive CRO). In the first set of models, commodity was included as a predictor, but commodity specific variables (meat cut and salmon variety) were excluded. In the second set of models, multivariable regression was performed for each commodity. For both sets of models the following procedures were followed: Variables were first screened with a univariate analysis using *p* ≤ 0.05, then we improved model performance by grouping United Nation-derived subregions (Table 1) into respective U.N-defined geographic regions (United Nations Statistic Division, 2022; e.g., North America, Latin America and the Caribbean, Asia, Europe, Oceania). We grouped “fresh” and “previously frozen” packaging as “sold at the counter.” Variables with low variability (>90% of the samples in one category) were excluded from the analysis; these included raised with/without antibiotics and tilapia meat cut. Samples with missing demographic data (e.g., “Unknown”) were also excluded. The final multivariable models were constructed using the stepwise variable selection procedure (*p* < 0.25 for a variable to enter the model and keeping only variables with a significance level *p* ≤ 0.05). All two-way interactions were evaluated. The direction and magnitude of the statistical associations were interpreted using the odds ratios (OR) with 95% CI.

**Table 1:** Epidemiologic information of seafood samples.

	Salmon *N* = 710 *n* (%)	Shrimp *N* = 710 *n* (%)	Tilapia *N* = 214 *n* (%)	Total seafood samples *N* = 1,634 *n* (%)
**Region of origin**				
Latin America and the Caribbean^1^	251 (35)	72 (10)	82 (38)	405 (25)
Northern America^2^	255 (36)	117 (17)	4 (1.9)	376 (23)
Eastern Asia^3^	84 (12)	2 (0.3)	102 (41)	188 (12)
South-eastern Asia^4^	2 (0.3)	335 (47)	10 (4.7)	347 (21)
Southern Asia^5^	-	116 (16)	-	116 (7.1)
Eastern Europe^6^	16 (2.3)	-	-	16 (1)
Northern and Western Europe^7^	30 (4.2)	1 (0.4)	-	31 (1.9)
Oceania^8^	1 (0.1)	-	-	1 (<1)
Unknown	68 (9.6)	65 (9.2)	16 (7.5)	149 (9.1)
United States plus Others^9^	3 (0.4)	1 (0.1)	-	4 (<1)
**Sold-as etc.**				
Sold as fresh	286 (40)	39 (5.5)	80 (37)	405 (25)
Previously Frozen	77 (11)	199 (28)	16 (7.5)	292 (18)
Frozen	332 (47)	460 (65)	117 (55)	909 (56)
Unknown	15 (2.1)	11 (1.6)	16 (7.4)	42 (2.6)
**Raising claims (antibiotic use)**				
None	691 (97)	689 (97)	214 (100)	1,594 (98)
No antibiotics ever/organic	19 (2.4)	20 (2.8)	-	39 (2)
**Raising claims (farming practice)**				
Wild-caught	338 (48)	176 (25)	1 (0.5)	515 (32)
Farm-raised	322 (45)	476 (67)	198 (93)	996 (61)
Unknown	50 (7)	56 (8)	15 (7)	121 (7.4)
Other	-	1 (0.1%)		1 (<1)
**Meatcut**				
Fillet	637 (91)	-		
Whole	-	-		
Steak	27 (3.9)	-		
Other	35 (5.0)	-		
Head-on/shell-on	-	91 (13)		
Peeled/deveined	-	32 (4.5)		
Peeled/tail on	-	96 (13.5)		
Peeled/undeveined	-			
Shell-on/headless	-	357 (50)		
Other	-	134 (19)		
**Salmon variety**				
Atlantic	274 (40)	-		
Sockeye	157 (23)	-		
Other	154 (23)	-		
Unknown	99 (15)	-		

1 Includes samples from Argentina, Brazil, Chile, Colombia, Costa Rica, Ecuador, Honduras. Mexico, Panama, Peru, Venezula, and any combination of the above.

2 Includes samples from Canada and the United States.

3 Includes samples from China and Taiwan.

4 Includes samples from Indonesia, Malaysia, Thailand, and Vietnam.

5 Includes samples from Bangladesh and India.

6 Includes samples from Poland and Russia.

7 Includes samples from Northern (Denmark, Iceland, Norway, United Kingdom or any combination of the above) and Western Europe (Germany).

8 Includes New Zealand.

9 Includes samples from China and United States, Honduras and United States, Norway and United States, Chile and United States.

## Results

### Prevalence of Bacterial Contamination Among Sampled Seafood

**Figure 1:**
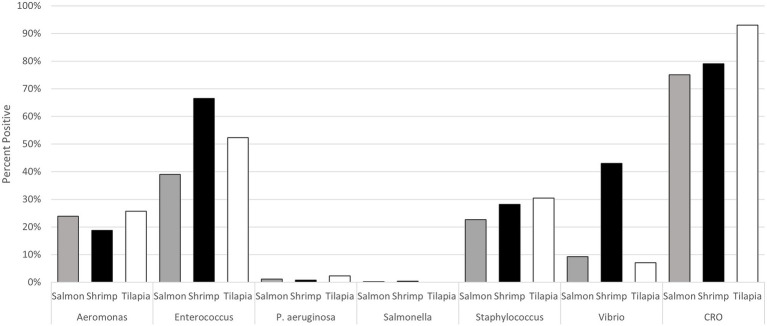
Prevalence of the target bacterial genera and presumptive carbapenem resistant microorganisms in the seafood samples collected from retail food stores in eight states in 2019.

Of the bacteria of interest, the predominant genus in all seafood samples was *Enterococcus* (Figure 1). Approximately 66% of shrimp samples were positive for this genus, as well as 52% of tilapia samples and 39% of salmon samples collected. *Salmonella* had the lowest prevalence, with one of 506 salmon samples yielding a *Salmonella* Reading and two of 498 shrimp samples yielding a *Salmonella* Teko and *Salmonella* Newport, respectively (Supplementary Table S1). No *Salmonella* were recovered from tilapia. Similarly, prevalence of *P. aeruginosa* was low in salmon (1.1%, 8/710), shrimp (0.9%, 6/710), and tilapia (2.3%, 5/214) samples (Figure 1). *Aeromonas* and *Staphylococcus* were present in ~20%–30% of all seafood samples. *Vibrio* had the most variable prevalence across the commodities, with 41% (290/710) of shrimp samples yielding isolates of this genus versus 9.0% (64/710) of salmon and 7.0% (14/214) of tilapia samples. Approximately 75% of salmon, 79% of shrimp, and 93% of tilapia samples yielded at least one presumptive CRO isolate. We identified 28 different genera among the 948 presumptive CRO isolates tested, with the most predominate being *Pseudomonas* (40% of all the isolates), followed by *Stenotrophomonas* and *Acinetobacter* (9.54% each), *Serratia* (8.5%), and *Aeromonas* (8.0%; Table 2).

**Table 2:** Prevalence of bacterial genera among presumptive CRO.

Organism	Total no. of isolates (%)
*Pseudomonas*	376 (39.7)
*Stenotrophomonas maltophilia*	90 (9.5)
*Acinetobacter*	90 (9.5)
*Serratia*	81 (8.5)
*Aeromonas*	76 (8.0)
Unidentified	50 (5.3)
*Morganella*	29 (3.1)
*Myroides* spp.	21 (2.2)
*Vibrio*	19 (2.0)
*Enterobacter*	19 (2.0)
*Citrobacter*	19 (2.0)
*Shewanella*	17 (1.8)
*Proteus*	17 (1.8)
*Sphingomonas*	16 (1.7)
*E. coli*	4 (0.4)
*Pantoea*	3 (0.3)
*Kluyvera*	3 (0.3)
*Raoultella ornithinolytica*	2 (0.2)
*Klebsiella*	2 (0.2)
*Cronobacter sakazakii* group	2 (0.2)
*Chryseobacterium* spp.	2 (0.2)
*Buttiauxela agrestis*	2 (0.2)
*Alcaligenes faecalis*	2 (0.2)
*Yersinia*	1 (0.1)
*Hafnia alvei*	1 (0.1)
*Delftia acidovorans*	1 (0.1)
*Cupriavidus pauculus*	1 (0.1)
*Burkholderia cepacia* group	1 (0.1)
*Brevundimonas diminuta*	1 (0.1)

### Antimicrobial Resistance Prevalence

The prevalence of phenotypic AMR and distribution of antimicrobial drug MICs for the isolates are listed for the target bacteria in Tables 3, 4; Supplementary Figures 1–6. There was a consistently low prevalence of Gram-negative organisms that were classified as resistant. All three *Salmonella* were susceptible to all antimicrobials tested. For *P. aeruginosa*, four drugs (colistin, ciprofloxacin, gentamicin, and meropenem) have CLSI clinical breakpoints for human infection treatment (Clinical and Laboratory Standards Institute, 2019), and all isolates were susceptible to these four drugs. However, all of these isolates demonstrated MICs ≥ 32 μg/ml for amoxicillin-clavulanic acid, ampicillin, azithromycin, cefoxitin, chloramphenicol, and nalidixic acid, and ≥256 μg/ml for sulfisoxazole. Depending on the commodity, 27%–44% of *Vibrio* isolates, which are known to have some intrinsic β-lactam resistance mechanisms (Chiou et al., 2015), were resistant to ampicillin. Less than 7% of the *Vibrio* isolates exhibited resistance to other antimicrobials. Prevalence of resistance was low in *Aeromonas* isolates (8%; Table 5).

**Table 3:** Percent resistance (%R) among Gram-negative seafood isolates.

Antimicrobials	Commodity	*Salmonella* %R	*Aeromonas* spp. %R	*Vibrio* spp. %R	*P. aeruginosa* %R
Amoxicillin-Clavulanic Acid	Salmon	0.0		0.0	
	Shrimp	0.0		2.2	
	Tilapia			0.0	
Ampicillin	Salmon	0.0		42.1	
	Shrimp	0.0		43.3	
	Tilapia			26.7	
Azithromycin	Salmon	0.0			
	Shrimp	0.0			
	Tilapia				
Cefoxitin	Salmon	0.0	2.9	0.0	
	Shrimp	0.0	1.6	1.7	
	Tilapia		4.2	0.0	
Ceftriaxone	Salmon	0.0	0.0		
	Shrimp	0.0	1.6		
	Tilapia		0.0		
Chloramphenicol	Salmon	0.0	0.0	0.0	
	Shrimp	0.0	0.0	0.0	
	Tilapia		0.0	0.0	
Ciprofloxacin	Salmon	0.0	0.0	0.0	0.0
	Shrimp	0.0	0.0	0.6	0.0
	Tilapia		0.0	0.0	0.0
Colistin	Salmon	0.0			0.0
	Shrimp	0.0			0.0
	Tilapia				
Gentamicin	Salmon	0.0	0.0	0.0	0.0
	Shrimp	0.0	0.0	1.1	0.0
	Tilapia		0.0	0.0	0.0
Meropenem	Salmon	0.0	0.0	0.0	0.0
	Shrimp	0.0	0.0	1.7	0.0
	Tilapia		0.0	0.0	0.0
Nalidixic Acid	Salmon	0.0			
	Shrimp	0.0			
	Tilapia				
Sulfisoxazole	Salmon	0.0		0.0	
	Shrimp	0.0		0.0	
	Tilapia			0.0	
Tetracycline	Salmon	0.0	2.9	0.0	
	Shrimp	0.0	8.2	5.5	
	Tilapia		0.0	0.0	
Trimethoprim-Sulfamethoxazole	Salmon	0.0	0.0	0.0	
	Shrimp	0.0	1.6	0.6	
	Tilapia		0.0	0.0	

Blank spaces indicate breakpoints do not exist for this organism-drug combination or a source was not tested. The number of isolates tested against each antimicrobial (except colistin) are as follows: *Salmonella* (Salmon, *N* = 1; Shrimp, *N* = 2; Tilapia were not tested); *Aeromonas* (Salmon, *N* = 70; Shrimp, *N* = 61; Tilapia, *N* = 48); *Vibrio* (Salmon, *N* = 17; Shrimp, *N* = 178; Tilapia, *N* = 15); *P. aeruginosa* (Salmon, *N* = 8; Shrimp, *N* = 6; Tilapia, *N* = 5). The number of isolates tested against colistin were: *Salmonella* (Salmon, N = 1; Shrimp, N = 2; Tilapia were not tested); *Aeromonas* (Salmon, N = 8; Shrimp, N = 6; Tilapia were not tested); *Vibrio* (Salmon, *N* = 5; Shrimp, *N* = 33; Tilapia, *N* = 4); *P. aeruginosa* (Salmon, *N* = 4; Shrimp, *N* = 2; Tilapia were not tested). MIC distributions and additional information are available in Supplementary Figures 1–4.

**Table 4:** Percent resistance (%R) among Gram-positive seafood isolates.

Antimicrobials	Commodity	*Enterococcus* spp. %R	*Staphylococcus* spp. %R
Ampicillin	Salmon	0.7	
	Shrimp	0.5	
	Tilapia	0.0	
Avilamycin	Salmon	1.5	
	Shrimp	0.0	
	Tilapia	0.0	
Chloramphenicol	Salmon	3.0	1.4
	Shrimp	1.0	0.0
	Tilapia	6.9	3.1
Ciprofloxacin	Salmon	3.0	0.0
	Shrimp	0.5	0.0
	Tilapia	0.0	0.0
Daptomycin	Salmon	0.7	26.8
	Shrimp	1.6	31.6
	Tilapia	0.0	72.3
Erythromycin	Salmon	6.0	1.4
	Shrimp	3.1	0.0
	Tilapia	13.8	1.5
Gentamicin	Salmon	0.7	0.0
	Shrimp	0.0	0.0
	Tilapia	0.0	0.0
Linezolid	Salmon	2.2	0.0
	Shrimp	1.0	0.0
	Tilapia	1.7	1.5
Nitrofurantoin	Salmon	1.5	0.0
	Shrimp	0.0	0.0
	Tilapia	3.4	0.0
Quinupristin-Dalfopristin	Salmon	1.5	1.4
	Shrimp	1.6	1.3
	Tilapia	6.9	1.5
Streptomycin	Salmon	3.7	
	Shrimp	2.6	
	Tilapia	6.9	
Tetracycline	Salmon	23.9	1.4
	Shrimp	12.4	0.0
	Tilapia	24.1	7.7
Tigecycline	Salmon	1.5	
	Shrimp	1.0	
	Tilapia	0.0	
Vancomycin	Salmon	1.5	0.0
	Shrimp	0.5	0.0
	Tilapia	0.0	1.5

Blank spaces indicate breakpoints do not exist for this organism-drug combination or a source was not tested. The number of isolates tested against each antimicrobial (except quinupristin/dalfopristin) are as follows: *Enterococcus* (Salmon, *N* = 134; Shrimp, *N* = 193; Tilapia, *N* = 58); *Staphylococcus* (Salmon, *N* = 71; Shrimp, *N* = 74; Tilapia, *N* = 65); For quinupristin/dalfopristin, testing results are shown for confirmed *E. faecium* only: Salmon, *N* = 5; Shrimp, *N* = 4; Tilapia, *N* = 7. MIC distributions and additional information are available in Supplementary Figures 5, 6.

**Table 5:** Number (*n*) of isolates with resistance to at least 1 antimicrobial.

	Salmon	Shrimp	Tilapia
	(*n*/total no. of isolates)	(*n*/total no. of isolates)	(*n*/total no. of isolates)
*Aeromonas*	4/70	6/61	2/48
*Enterococcus*	35/134	27/193	13/58
*Pseudomonas*	0/8	0/6	0/5
*Salmonella*	0/1	0/2	-
*Staphylococcus*	2/71	0/74	2/65
*Vibrio*	0/17	0/178	0/15
Total no. of isolates resistant to at least 1 antimicrobial	41	33	17

Among the Gram-positive organisms, *Enterococcus* spp. exhibited the highest prevalence of resistance, with the most common resistance to tetracycline (12%–24%), followed by erythromycin (3%–14%). *Enterococcus faecium* were also commonly resistant to quinupristin/dalfopristin, with the resistance prevalence ranging from 40% to 75%, depending on source. Prevalence of resistance in enterococci from tilapia was two to seven times higher than that in the isolates from shrimp or salmon for each chloramphenicol, erythromycin, nitrofurantoin, quinupristin-dalfopristin (*E. faecium* only), and streptomycin. Prevalence of tetracycline resistance in enterococci from tilapia and salmon was two times higher than that in isolates from shrimp. However, all enterococci from tilapia were susceptible to ampicillin, avilamycin, ciprofloxacin, daptomycin, gentamicin, tigecycline, and vancomycin. We found two enterococci from salmon that were resistant to avilamycin, an orthosomycin antimicrobial intended for use only in broiler chickens and weaner pigs. One of the isolates came from a product imported from Chile and the other from a product imported from Canada (Supplementary Table S1). Prevalence of daptomycin resistance in staphylococci from tilapia was 2 to 3 times higher than that found in isolates from shrimp or salmon. Less than 8% of all the *Staphylococcus* isolates from all sources were resistant to the other antimicrobials tested. Table 6 shows patterns of multidrug resistance (MDR, resistant to ≥3 classes), which was overall very low. Approximately 2% (21/1006) of isolates tested were MDR.

**Table 6:** MDR patterns in bacterial isolates from seafood.

Genus	Commodity	CVM number	Resistance pattern
*Enterococcus*	Salmon	SP19E00016	AMP-AVL-CHL-DAP-ERY-LZD-NIT-VAN
	Salmon	SP19E00130	AVL-ERY-LZD-NIT-TGC-VAN
	Shrimp	SP19E00345	DAP-ERY-LZD-TET-TGC-VAN
	Tilapia	SP19E00935	CHL-ERY-LZD-NIT-QDA-TET
	Salmon	SP19E00274	CIP-ERY-STR-TET
	Shrimp	SP19E00120	CIP-ERY-STR-TET
	Shrimp	SP19E00346	DAP-LZD-TET-TGC
	Tilapia	SP19E00519	ERY-NIT-STR-TET
	Salmon	SP19E00158	CHL-STR-TET
	Salmon	SP19E00177	CHL-ERY-TET
	Salmon	SP19E00399	ERY-GEN-TET
	Shrimp	SP19E00023	ERY-STR-TET
	Shrimp	SP19E00214	DAP-STR-TET
	Shrimp	SP19E00364	CHL-ERY-TET
	Tilapia	SP19E00686 SP19E00689	ERY-STR-TET
	Tilapia	SP19E00083	CHL-ERY-TET
*Aeromonas*	Shrimp	SP19A00185	FOX-TET-COT
*Staphylococcus*	Tilapia	SP19ST00584	CHL-DAP-ERY-LZD-QDA-TET-VAN
	Tilapia	SP19ST00359	CHL-DAP-TET
*Vibrio*	Shrimp	SP19V00074	AMC-AMP-FOX-MER
	Shrimp	SP19V00235	AMC-AMP-FOX-COT

AMC, amoxicillin-clavulanic acid; AMP, ampicillin; AVL, avilamycin; CHL, chloramphenicol; CIP, ciprofloxacin; COT, trimethoprim-sulfamethoxazole; DAP, daptomycin; ERY, erythromycin; FOX, cefoxitin; GEN, gentamicin; LZD, linezolid; MER, meropenem; NIT, nitrofurantoin; QDA, quinupristin-dalfopristin; STR, streptomycin; TET, tetracycline; TGC, tigecycline; and VAN, vancomycin.

### Risk Factor Analysis

Epidemiological risk factors (geographic region of origin, claims about antibiotic use, farm raising claims, and “sold as”) were analyzed for their association with isolation of at least one of the targeted bacterial genera (Tables 7, 8) and for association with the isolate resistance to at least one of the tested antimicrobials (Table 9). In the final multivariable logistic regression model, four epidemiologic risk factors were significantly associated with the sample contamination with one of the targeted bacterial genera: the sample being shrimp vs. salmon, originating from Asia or Latin America and the Caribbean vs. North America, and sold at the counter vs. purchased frozen. Looking at each seafood commodity independently, we found that for salmon, an Asian country of origin and counter purchasing increased the odds of bacterial contamination by 3.4- and 2.6-fold, respectively. A farm raising claim was the only variable significantly associated with bacterial contamination of a shrimp sample. The odds of recovery from farm-raised shrimp were 1.9 times higher than wild-caught shrimp.

**Table 7:** Adjusted ORs for growth of at least one bacterium in all seafood samples. Risk factors that did not fit the model (i.e., farm raising claim) are not shown. (*n*=) is the number of samples analyzed.

	OR (95% CI)
**Source**	
Salmon (*n* = 710)	ref
Shrimp (*n* = 710)	**2.8 (2.0–3.9)**
Tilapia (*n* = 214)	1.2 (0.8–1.9)
**Region of origin**	
North America (*n* = 376)	ref
Asia (*n* = 653)	**2.7 (1.8–3.7)**
Europe (*n* = 47)	2.1 (1.0–4.2)
Latin America and the Caribbean (*n* = 406)	**1.6 (1.1–2.3)**
**Sold as, etc.**	
Sold at the counter (*n* = 700)	**2.1 (1.6–2.9)**
Frozen (*n* = 653)	ref

Bold values are statistically significant.

**Table 8:** Adjusted ORs for growth of at least one target bacterium in each commodity.

	Salmon OR (95% CI)	Shrimp OR (95% CI)	Tilapia OR (95% CI)
**Region of origin**			
North America	ref		
Asia	**3.4 (1.9–6.1)**		
Europe	2.0 (1.0–4.2)		
Latin America and the Caribbean	1.4 (0.9–2.2)		
**Sold as, etc.**			
Sold at the counter	**2.6 (1.7–3.9)**	1.8 (1.0–3.2)	
Frozen	ref	ref	
**Raising claim (farming practice)**			
Wild caught		ref	n/a
Farm-raised		**1.9 (1.2–3.2)**	n/a

Blank cells indicate risk factor did not fit the model for that commodity. Risk factors that did not fit any models (i.e., shrimp and salmon meat cuts and salmon variety) are not shown. n/a, not applicable; risk factor was not evaluated for this commodity. Bold values are statistically significant.

**Table 9:** Adjusted OR for resistance to at least antimicrobial. Risk factors that did not fit the model (i.e., region of origin and “sold-as”) are not shown. (*n*=) is the number of samples analyzed.

	OR (95% CI)
**Source**	
Salmon (*n* = 710)	ref
Shrimp (*n* = 710)	**0.5 (0.3–0.9)**
Tilapia (*n* = 214)	0.9 (0.4–1.8)
**Raising claim (farming practice)**	
Wild-caught (*n* = 515)	ref
Farm-raised (*n* = 999)	**0.4 (0.3–0.7)**

Bold values are statistically significant.

Due to low a prevalence of resistance overall in seafood, we examined the association of epidemiologic factors with resistance to at least one of the tested antimicrobials among the target genera (i.e., not including presumptive CRO). Using the full multivariable model with contamination of a sample with an antimicrobial resistant isolate of the target bacterial genera as the dependent variable, we found that shrimp were less resistant than salmon (OR 0.5, CI 0.3–0.9), and that farm-raised shrimp and salmon were 60% less likely to yield a resistant isolate. This odds ratio was most likely driven by a larger proportion of tetracycline and daptomycin-resistant isolates from wild-caught commodities (Supplementary Figure 7). However, there was greater diversity in resistance phenotypes among the isolates from farm-raised commodities.

### ARGs

Thirty-three percent (26/79) of the non-CRO, non-*Salmonella* isolates that were sequenced carried no known resistance genes despite having phenotypic resistance to at least one of the antimicrobials tested (Supplementary Table S1). Similarly, 34% (98/288) of presumptive CRO isolates did not harbor any known ARGs even though they all exhibited decreased phenotypic susceptibility to carbapenems (as defined by the growth on carbapenem containing agar; Supplementary Table S1). Intrinsic or other as-yet-to-be-annotated genes were likely responsible for the phenotypic resistance in isolates without known ARGs. Table 10 lists the 156 unique ARGs identified from the 370 strains. A number of presumptive CRO had carbapenemase genes that are normal constituents of those bacteria, including *bla*_L1_ in *Stenotrophomonas*, *cphA*/*cphA1* in *Aeromonas*, and members of the *bla*_OXA-51_-like and *bla*_OXA-213_-like families in *Acinetobacter.* We identified three isolates with carbapenemases that are known to transfer horizontally. An isolate each of *Aeromonas* spp. and an *Acinetobacter baumanii* each harbored the *bla*_NDM-1_ gene, and an isolate of *Enterobacter cloacae* carried the *bla*_IMI-2_ gene. All three isolates were recovered from shrimp imported from Southeast Asia. At least 76% of the 37 cephalosporinases identified were also common in the bacterial genera in which detected, including chromosomally encoded inducible AmpC β-lactamases in *Pseudomonas* (*bla*_PDC_ alleles; Rodríguez-Martínez et al., 2009) and *Acinetobacter* (*bla*_ADC_ alleles; Hujer et al., 2005; Gordon and Wareham, 2010). We found *mcr-3.3* in an *Aeromonas* isolated from CRO media, however the MIC was only 0.5 μg/ml. Other studies have shown that colistin susceptibility is determined by whether *mcr-3.3* is located on the chromosome or a plasmid (Ling et al., 2017; Shen et al., 2018). We identified one extended spectrum β-lactamase (ESBL) gene, *bla*_CTX-M-55_, in an *Aeromonas* isolated from CIN media. The gene conferred resistance to ampicillin and ceftriaxone (Supplementary Table S1). The isolate also contained *qnrS1,* a combination seen before on an IncF plasmid in isolates from NARMS retail meat surveillance (Tyson et al., 2019). Although *qnrS1* confers resistance to quinolones and reduced susceptibility to fluroquinolones in *E. coli* and *Salmonella*, this *Aeromonas* isolate was susceptible to ciprofloxacin (MIC = 0.015 μg/ml). Other genotype–phenotype comparisons were difficult to make given the lack of accepted interpretive criteria to categorize the phenotypic antimicrobial susceptibility of the isolates and the limited number of isolates sequenced, however some valuable information might be gleaned from the data. We found *qnrVC* genes in *Vibrio* susceptible to ciprofloxacin as well as tetracycline resistance genes, *tet(34)* and *tet(35)*, in isolates susceptible to the drug (Supplementary Table S1).

**Table 10:** AMR genes detected based on whole genome sequencing and annotation of the bacterial isolates^1^ from seafood samples.

Drug class (no. of isolates)	Subclass	ARGs	Organism	No. of isolates
Aminoglycoside (58)	Amikacin/Kanamycin (1)	*aph(3′)-IIIa*	*Staphylococcus*	1
	Aminoglycoside (14)	*aac(6′)*	*Stenotrophomonas*	1
		*aac(6′)-Iz*	*Stenotrophomonas*	1
		*aph(6)*	*Stenotrophomonas*	13
	Gentamicin (4)	*aac(6′)-IIa*	*Delftia acidovorans*	1
			*Vibrio*	1
		*ant(2″)-Ia*	*Acinetobacter*	1
			*Aeromonas*	1
			*Vibrio*	1
	Kanamycin (16)	*aph(3′)-IIb*	*Pseudomonas*	5
		*aph(3′)-IIc*	*Stenotrophomonas*	11
	Streptomycin (36)	*aadA1*	*Aeromonas*	2
			*D. acidovorans*	1
			*Unidentified*	1
		*ant(3″)-IIa*	*Acinetobacter*	28
		*aph(3″)-Ib*	*Acinetobacter*	2
			*Aeromonas*	2
			*D. acidovorans*	1
			*Pseudomonas*	1
			*Vibrio*	1
		*aph(6)-Id*	*Acinetobacter*	2
			*Aeromonas*	2
			*D. acidovorans*	1
			*Vibrio*	1
	Tobramycin (1)	*aac(6′)-Iz*	*Stenotrophomonas*	1
β-Lactam (222)	β-Lactam (121)	*ampC*	*Aeromonas*	7
			*Enterobacter*	1
			*Serratia*	20
		*bla*_CARB_	*Vibrio*	1
		*bla*_CARB-7_	*Vibrio*	1
		*bla*_CARB-18_	*Vibrio*	9
		*bla*_CARB-20_	*Vibrio*	3
		*bla*_CARB-21_	*Vibrio*	2
		*bla*_CARB-42_	*Vibrio*	2
		*bla*_CMH_	*Enterobacter*	1
		*bla*_FONA_	*Aeromonas*	1
			*Pseudomonas*	1
			*Serratia*	14
		*bla*_FONA-1_	*Serratia*	1
		*bla*_FONA-4_	*Pseudomonas*	1
		*bla*_GIL_	*Citrobacter*	2
		*bla*_I_	*Staphylococcus*	4
		*bla*_L2_	*Stenotrophomonas*	2
		*bla*_OXA_	*Acinetobacter*	21
			*Aeromonas*	10
			*Pseudomonas*	3
			*Shewanella*	6
		*bla*_OXA-396_	*Pseudomonas*	1
		*bla*_OXA-494_	*Pseudomonas*	1
		*bla*_OXA-847_	*Pseudomonas*	1
		*bla*_PSE_	*Vibrio*	1
		*bla*_R1_	*Staphylococcus*	4
		*bla*_Z_	*Staphylococcus*	4
		*hugA*	*Citrobacter*	1
			*Unidentified*	1
	Carbapenem (100)	*bla*_IMI-2_	*Enterobacter*	1
		*bla*_IND_	*Chryseobacterium spp*	1
		*bla*_L1_	*Stenotrophomonas*	14
		*bla*_MUS_	*Myroides spp*	1
		*bla*_NDM-1_	*Acinetobacter*	1
			*Aeromonas*	1
		*bla*_OXA-51_ ^2^	*Acinetobacter*	1
		*bla*_OXA-64_ ^2^	*Acinetobacter*	1
		*bla*_OXA-68_ ^2^	*Acinetobacter*	1
		*bla*_OXA-69_ ^2^	*Acinetobacter*	1
		*bla*_OXA-91_ ^2^	*Acinetobacter*	1
		*bla*_OXA-98_ ^2^	*Acinetobacter*	1
		*bla*_OXA-106_	*Acinetobacter*	3
		*bla*_OXA-117_ ^2^	*Acinetobacter*	1
		*bla*_OXA-121_ ^2^	*Acinetobacter*	3
		*bla*_OXA-272_ ^3^	*Acinetobacter*	1
		*bla*_OXA-273_ ^3^	*Acinetobacter*	4
		*bla*_OXA-305_ *^3^*	*Acinetobacter*	3
		*bla*_OXA-402_ *^2^*	*Acinetobacter*	4
		*bla*_OXA-417_ ^3^	*Acinetobacter*	2
		*bla*_OXA-500_ ^3^	*Acinetobacter*	1
		*bla*_OXA-506_ ^3^	*Acinetobacter*	1
		*bla*_OXA-508_ ^2^	*Aeromonas*	1
		*bla*_OXA-685_ ^2^	*Acinetobacter*	1
		*bla*_OXA-820_ ^3^	*Acinetobacter*	2
		*bla*_OXA-821_ ^3^	*Acinetobacter*	1
		*bla*_POM-1_	*Aeromonas*	2
		*bla*_SPR_	*Serratia*	1
		*bla*_TRU_	*Aeromonas*	2
		*bla*_TUS_	*Myroides spp*	1
		*cphA*	*Aeromonas*	16
		*cphA1*	*Aeromonas*	27
			*Citrobacter*	1
			*Pseudomonas*	2
β-Lactam (continued)	Cephalosporin (79)	*bla*_ACC-1a_	*Hafnei*	1
		*bla*_ACT-16_	*Enterobacter*	1
		*bla*_ADC_	*Acinetobacter*	26
		*bla*_ADC-6_	*Acinetobacter*	1
		*bla*_ADC-12_	*Acinetobacter*	1
		*bla*_ADC-18_	*Acinetobacter*	3
		*bla*_ADC-23_	*Acinetobacter*	1
		*bla*_ADC-43_	*Acinetobacter*	2
		*bla*_ADC-50_	*Acinetobacter*	1
		*bla*_ADC-52_	*Acinetobacter*	1
		*bla*_ADC-57_	*Acinetobacter*	1
		*bla*_ADC-70_	*Acinetobacter*	1
		*bla*_ADC-76_	*Acinetobacter*	1
		*bla*_ADC-132_	*Acinetobacter*	4
		*bla*_ADC-135_	*Acinetobacter*	3
		*bla*_ADC-155_	*Acinetobacter*	1
		*bla*_ADC-163_	*Acinetobacter*	3
		*bla*_ADC-165_	*Acinetobacter*	1
		*bla*_ADC-169_	*Acinetobacter*	4
		*bla*_CMY_	*Citrobacter*	6
		*bla*_CMY-70_	*Citrobacter*	1
		*bla*_CMY-82_	*Citrobacter*	1
		*bla*_CMY-83_	*Citrobacter*	1
		*bla*_CTX-M-55_	*Aeromonas*	1
		*bla*_DHA_	*Staphylococcus*	1
		*bla*_FOX_	*Aeromonas*	1
		*bla*_MOX_	*Aeromonas*	1
		*bla*_PDC_	*Pseudomonas*	1
		*bla*_PDC-3_	*Pseudomonas*	1
		*bla*_PDC-45_	*Pseudomonas*	1
		*bla*_PDC-66_	*Pseudomonas*	1
		*bla*_PDC-109_	*Pseudomonas*	1
		*bla*_PDC-121_	*Pseudomonas*	1
		*bla*_RSC1_	*Burkholderia cepacia group*	1
			*Pseudomonas*	1
		*bla*_VEB-1_	*Vibrio*	1
	Methicillin (3)	*mecA1*	*Staphylococcus*	2
			*Unidentified*	1
	Bleomycin (1)	*ble*	*Acinetobacter*	1
	Colistin (1)	*mcr-3.3*	*Aeromonas*	1
	Fluoroquinolone (4)	*crpP*	*Pseudomonas*	4
	Fosfomycin (8)	*fosA*	*Enterobacter*	1
			*Pseudomonas*	6
		*fosB*	*Staphylococcus*	1
	Fusidic Acid (1)	*fusD*	*Staphylococcus*	1
	Lincosamide (1)	*lnu(A)*	*Macrococcus*	1
	Lincosamide/Streptogramin (5)	*lsa(A)*	*Enterococcus*	2
		*sal(A)*	*Staphylococcus*	2
			*Unidentified*	1
	Macrolide (3)	*erm(B)*	*Macrococcus*	1
		*erm(C)*	*Staphylococcus*	1
		*mph(E)*	*Acinetobacter*	1
		*msr(E)*	*Acinetobacter*	1
	Phenicol (13)	*catA*	*Staphylococcus*	1
		*catA2*	*Staphylococcus*	1
		*catB*	*D. acidovorans*	1
		*catB7*	*Pseudomonas*	6
		*catB9*	*Vibrio*	1
		*cmlA5*	*Aeromonas*	1
		*floR*	*Acinetobacter*	1
			*Aeromonas*	1
			*Vibrio*	1
	Phenicol/Quinolone (7)	*oqxA*	*Enterobacter*	2
β-Lactam (continued)			*Cronobacter*	2
		*oqxB*	*Enterobacter*	2
			*Escherichia*	2
			*Pseudomonas*	1
			*Cronobacter*	2
	Quinolone (27)	*qnrA3*	*Acinetobacter*	1
			*Pseudomonas*	1
			*Shewanella*	6
		*qnrB*	*Citrobacter*	1
		*qnrB9*	*Citrobacter*	1
		*qnrB17*	*Citrobacter*	1
		*qnrD*	*Vibrio*	1
		*qnrD1*	*Staphylococcus*	1
		*qnrE*	*Aeromonas*	1
		*qnrS1*	*Aeromonas*	1
		*qnrVC*	*Vibrio*	3
		*qnrVC1*	*Vibrio*	3
		*qnrVC4*	*Vibrio*	1
		*qnrVC6*	*Vibrio*	6
	Sulfonamide (11)	*sul1*	*Aeromonas*	3
		*sul2*	*Acinetobacter*	5
			*Aeromonas*	1
			*D. acidovorans*	1
			*Vibrio*	1
	Tetracycline (41)	*tet(34)*	*Vibrio*	18
		*tet(35)*	*Vibrio*	17
		*tet(38)*	*Staphylococcus*	3
		*tet(39)*	*Acinetobacter*	1
		*tet(A)*	*Aeromonas*	2
		*tet(B)*	*Acinetobacter*	1
			*Vibrio*	4
		*tet(D)*	*Staphylococcus*	1
		*tet(E)*	*Aeromonas*	7
			*Pseudomonas*	2
		*tet(H)*	*Citrobacter*	1
		*tet(K)*	*Staphylococcus*	2
		*tetA(D)*	*D. acidovorans*	1
	Trimethoprim (12)	*dfrA1*	*Aeromonas*	1
		*dfrA6*	*Vibrio*	3
		*dfrA7*	*Aeromonas*	1
		*dfrA15*	*Aeromonas*	1
β-Lactam (continued)		*dfrA16*	*D. acidovorans*	1
		*dfrA31*	*Vibrio*	3
		*dfrE*	*Enterococcus*	2
			*Unidentified*	1
		*dfrG*	*Staphylococcus*	1

1 If bacterial genera could not be resolved through *in silico* methods, VITEK identification was used.

2 Members of *bla*OXA-51 like family.

3 Members of *bla*OXA-213 like family (Evans and Amyes, 2014; National Center for Biotechnology Information, 2022)

## Discussion

We examined the prevalence of several target bacterial genera in retail seafood as well as their resistance attributes. We found remarkably lower levels of *Salmonella* contamination (<1%) than was found in another large scale FDA survey of local and imported seafood collected from processors and distributors throughout the United States (Heinitz et al., 2000). In that 2000 study, 11.8% of raw fin fish/skin fish and 8.9% of raw crustaceans were positive for *Salmonella*. Although both the Heinitz study and our study used FDA-BAM methods to isolate *Salmonella*, Heinitz et al. used composite samples from 15 cases of product, likely resulting in greater prevalence of bacteria. Additionally, the majority of *Salmonella* were isolated from imports, which represented a higher proportion of samples than in our study, and may have also impacted prevalence. Finally, federal regulations or guidance published in the intervening period may have had some impact on *Salmonella* contamination. Levels of *Salmonella*, *Aeromonas*, *Vibrio*, and *Enterococcus* we found were more consistent with other studies (Tuševljak et al., 2012; Jans et al., 2018). *Vibrio* and *Aeromonas* were the most frequently isolated Gram-negative bacteria and *Enterococcus* were the most frequently isolated Gram-positive bacterium, making them potentially better candidates for tracking AMR. However, there is a need to establish internationally accepted MIC breakpoints (i.e., epidemiological cut-off values) for many drugs with activity against *Vibrio* and *Aeromonas* in order to track trends over time. There has been some progress in this area (Baron et al., 2017).

Shrimp had the highest odds of recovery of our target bacteria. Given its high consumption rate in the United States, this would certainly be a top priority seafood commodity to include in a long-term monitoring or surveillance program. If resources allow, salmon and tilapia should also be included in a United States-focused monitoring or surveillance program because they are also highly consumed. These data suggest a need for tracking domestically produced and imported seafood to assess the potential for consumer exposure to antimicrobial resistant bacteria. Sample collection could include a higher proportion of samples from Asia and Latin America and the Caribbean, as these were more likely to produce the target bacteria. When available, fresh or previously frozen seafood—especially salmon, would be preferred over frozen samples as thawed and fresh commodities were more likely to yield our target bacteria. Based on the bacterial prevalence results from this pilot study, NARMS began monitoring seafood in 2020 to test *Vibrio*, *Enterococcus* and *Aeromonas* species and lactose fermenters in shrimp, salmon, and tilapia.

Targeted bacteria exhibited pan-susceptibility (*Salmonella* and *P. aeruginosa*) or low levels of resistance (<10%) to antimicrobials with interpretive criteria for these organisms, with the exception of ampicillin (*Vibrio*), daptomycin (*Staphylococcus*), quinupristin-dalfopristin (*E. faecium*) and tetracycline (*Enterococcus* spp.). While many *P. aeruginosa* isolates did have MIC values at or above 32 μg/ml for other antimicrobials including amoxicillin-clavulanic acid, ampicillin, and chloramphenicol, it is known that this organism harbors a high number of intrinsic resistance mechanisms (Pang et al., 2019). Similarly, the ampicillin and daptomycin resistance phenotypes in *Vibrio* and *Staphylococcus*, respectively, are ostensibly mediated by mechanisms intrinsic to those bacteria. Some species of *Vibrio* have been shown to harbor chromosomally encoded class A carbenicillin-hydrolyzing β lactamases (*bla*_CARB_; Chiou et al., 2015), conferring intrinsic resistance to ampicillin. The *bla*_CARB_ genes were present in all of *Vibrio* isolates sequenced for this study. Additionally, *Staphylococcus sciuri*, which comprised the majority of *Staphylococcus* spp. recovered, are intrinsically less susceptible to daptomycin (Sader and Jones, 2012; Schoenfelder et al., 2017) than other *Staphylococcus* species.

Resistance may also be mediated by acquisition of exogenous determinants as a result of antimicrobial selection pressure. Selection pressure from tetracyclines, which are approved for use in fish in the United States (U.S. Food and Drug Administration, 2020b) and are among the most frequently used and most consumed (in mg/kg) antimicrobials in aquatic food globally (Schar et al., 2020), and may have promoted the high levels of tetracycline resistance we observed in *Enterococcus*. Tetracyclines are not approved for use in shrimp produced in the United States (U.S. Food and Drug Administration, 2021), which may partially explain why tetracycline resistance levels were almost two-fold lower among enterococci from retail shrimp than retail salmon and tilapia. Streptogramins are not authorized for use in aquaculture in the United States, and there is little evidence of their use in other countries, therefore the presence of quinupristin-dalfopristin resistant *E. faecium* and *Staphylococcus* isolates remains to be investigated. There was no correlation between the presence of antimicrobial resistant bacteria and country of origin.

Although levels of resistance in *Enterococcus* were low overall, we did observe an appreciable difference between tilapia, salmon, and shrimp. There were no tilapia isolates resistant to tigecycline, vancomycin, gentamicin, daptomycin, or ampicillin, which may simply be due to a combination of the smaller number of isolates tested (*n* = 58), compared to salmon (*n* = 134) and shrimp (*n* = 193), and the low prevalence of resistance to these drugs overall. However, *Enterococcus* from tilapia had almost two-fold higher levels of resistance to chloramphenicol, erythromycin, nitrofurantoin, quinupristin-dalfopristin (*E. faecium* only), and streptomycin compared to isolates from shrimp or salmon, which could be a possible consequence of commodity-specific differences in the species composition of isolates with higher MICs. We observed a larger proportion of non-faecalis *Enterococcus* species among the resistant tilapia isolates (Supplementary Table S1) than among the resistant shrimp and salmon isolates. In contrast, species distribution of daptomycin resistant *Staphylococcus* isolates was similar across all commodities (Supplementary Table S1), despite tilapia isolates having two-fold higher levels of resistance to this drug compared to isolates from salmon and shrimp. A recent study suggests that tilapia receive more antimicrobials than shrimp and salmon (Schar et al., 2020). Indeed, 93% of tilapia collected in our study were farm-raised compared to just 67% of shrimp and 45% of salmon. However, the dosage and type of antimicrobials these animals receive are largely obscure due to inadequate statistics on antimicrobial usage in aquaculture. While one would assume that farm-raised shrimp and salmon would also be exposed to more antimicrobials and thus have a higher likelihood of harboring resistant bacteria than wild-caught seafood, that did not appear to be the case in our samples. We found that farm-raised shrimp and salmon had a 60% lower likelihood of containing resistant bacteria. This was in part explained by higher levels of resistance to tetracycline and daptomycin in bacteria from wild-caught shrimp and salmon, and also differential distribution of bacterial species among the raising claims (for example, *S. sciuri*, comprised 54% of *Staphylococcus* tested from wild-caught salmon, but only 17% of *Staphylococcus* from farm-raised salmon; Supplementary Figure 7). Notably, bacteria from farm-raised seafood were resistant to more types of antimicrobials than bacteria from wild caught seafood. Our results oppose those of others who have shown that farm raised shrimp are more abundant in ARGs compared to wild caught shrimp (Sharma et al., 2021). It is unclear why wild-caught shrimp and salmon in this study would harbor bacteria that are more resistant to these drugs, although a few possible scenarios could be at play including: exposure to sewage or agricultural pollution from farms, as-yet-unknown impacts from climate change and plastic pollution on coastal waters, and potential mislabeling of product.

Some bacteria considered to be hospital-acquired could have foodborne origins. Using selective media, we found carbapenem-resistant *Acinetobacter baumannii* in the aquatic environment from seafood that were both farm-raised and wild-caught. *Acinetobacter* are ubiquitous in the environment, and some *Acinetobacter* species, including *A. baumannii* are recognized as opportunistic fish pathogens (Dekić et al., 2018). Our findings suggest that *A. baumannii* could potentially be transmitted to humans through the seafood chain. Among the 55 *Acinetobacter* isolates that were sequenced, we identified 47 predicted genes, 36 of which were naturally occurring, including undescribed *bla*_ADC_ cephalosporinase and *bla*_OXA_ oxacillinase alleles. Other than *ant(3″)-IIa* spectinomycin resistance genes, few clinically relevant acquired ARGs were found, supporting previous evidence that environmental strains appear different from highly resistant clinical isolates associated with nosocomial environments (Klotz et al., 2019).

One limitation of the study was that we were unable to sequence all isolates presenting a non-wild-type phenotype. This put us at a disadvantage to compare genotypes with respective phenotypes. Additionally, we could not confirm whether the genetic mechanisms were intrinsic to the organism or horizontally acquired. We suppose that intrinsic mechanisms common to a bacterial species would be less affected by the variables we tested in our model, and our odds ratio may potentially overestimate the association of source and farm raising claim with AMR. Lastly, no detailed information on differences in aquaculture practices and facilities was available on the sample packaging to help explain the significant differences in the bacterial contamination prevalence and antimicrobial resistant bacteria prevalence between seafood commodities or seafood from different regions. Despite these limitations, we were able to complete a multi-lab study using standardized methods to assess a population-scale prevalence of resistant bacteria in seafood. Our results correlated with others showing that bacteria with clinically relevant carbapenamase genes can be found in imported seafood (Janecko et al., 2016). We also identified a number of genes that might have clinical relevance when expressed in zoonotic pathogens such as *Salmonella* but are intrinsic to the environmental aquatic bacteria in which they were discovered (Table 10). Additional work is needed to determine if these genes are located on plasmids or transposable elements within chromosomes, as these aquatic bacteria may act as reservoirs for clinically relevant ARGs, and contribute to their potential dissemination.

## Conclusion

Even though seafood has become an increasingly important source of animal protein for the United States general population over the past decade, it has been understudied in the area of foodborne AMR. Here, we have shown that while imported and domestically produced seafood can be contaminated with antimicrobial resistant bacteria, the prevalence of contamination with resistant bacteria is low. However, because antimicrobials are used in aquaculture it is worthwhile to continue monitoring these food commodities for resistant bacteria. Plans for continued monitoring must consider differences in AMR prevalence by seafood type, country of origin, and raising practices, and the presence of not-yet-identified ARGs in bacteria contaminating seafood. This seafood pilot study provides an important basis for the NARMS seafood surveillance that began in January 2020.

## Data Availability Statement

The datasets presented in this study can be found in online repositories. The names of the repository/repositories and accession number(s) can be found in the article/Supplementary Material.

## Author Contributions

HT, SA, EN, CW, GT, and PM contributed to the conception and design of the study. XL, MT-D’A, VV, RH, LM, NM’i, and LR conducted sampling. SA, SB, SY, CR-T, SS, XL, MT-D’A, VV, RH, LM, NM’i, LR, and SB-J all conducted microbial testing. CL conducted whole genome sequencing. CL and ES performed bioinformatic analysis. EN and HT performed statistical analysis. HT wrote the first draft of the manuscript. All authors contributed to the manuscript revision, read, and approved the submitted version.

## Funding

This work is supported by the United States Food and Drug Administration.

## Author Disclaimer

The views expressed in this article are those of the authors and do not necessarily reflect the official policy of the Department of Health and Human Services, the United States Food and Drug Administration, or the United States Government. The opinions expressed in this article are the author’s own and do not reflect the view of the National Institutes of Health. Reference to any commercial materials, equipment, or process does not in any way constitute approval, endorsement, or recommendation by the Food and Drug Administration.

## Conflict of Interest

The authors declare that the research was conducted in the absence of any commercial or financial relationships that could be construed as a potential conflict of interest.

## Publisher’s Note

All claims expressed in this article are solely those of the authors and do not necessarily represent those of their affiliated organizations, or those of the publisher, the editors and the reviewers. Any product that may be evaluated in this article, or claim that may be made by its manufacturer, is not guaranteed or endorsed by the publisher.

## Footnotes

^1^ https://www.ncbi.nlm.nih.gov/pathogens/antimicrobial-resistance/AMRFinder/

^2^ https://github.com/tseemann/mlst

^3^ https://pubmlst.org/
