# Supplementary figures and images for: Prevalence of Antimicrobial Resistance in Select Bacteria From Retail Seafood—United States, 2019
Source: Front Microbiol. 2022 Jun 23;13:928509. doi: 10.3389/fmicb.2022.928509 (PMC9262255; doi:10.3389/fmicb.2022.928509)

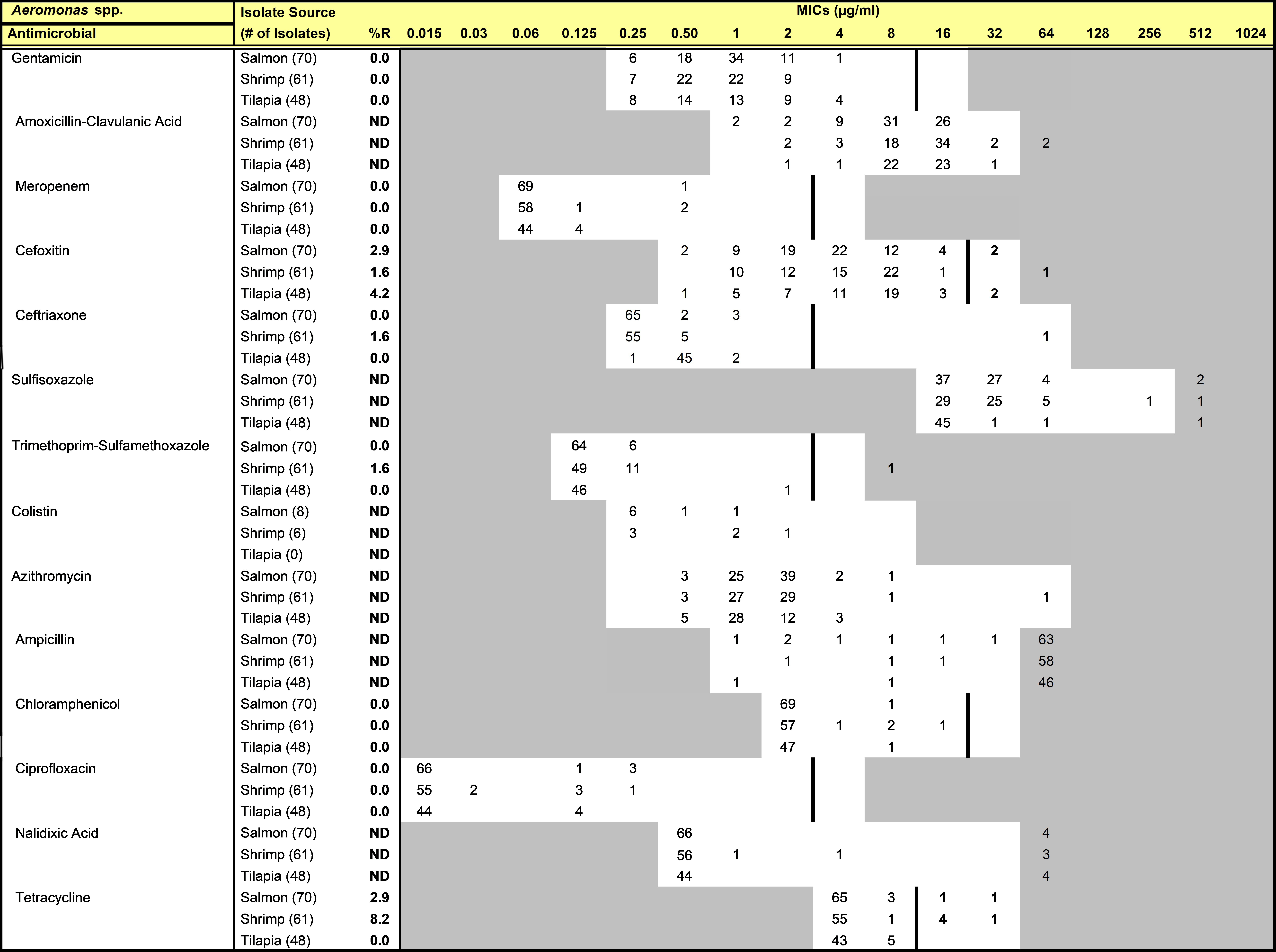

Supplement: Supplementary file 1 [file Image_1.JPEG]

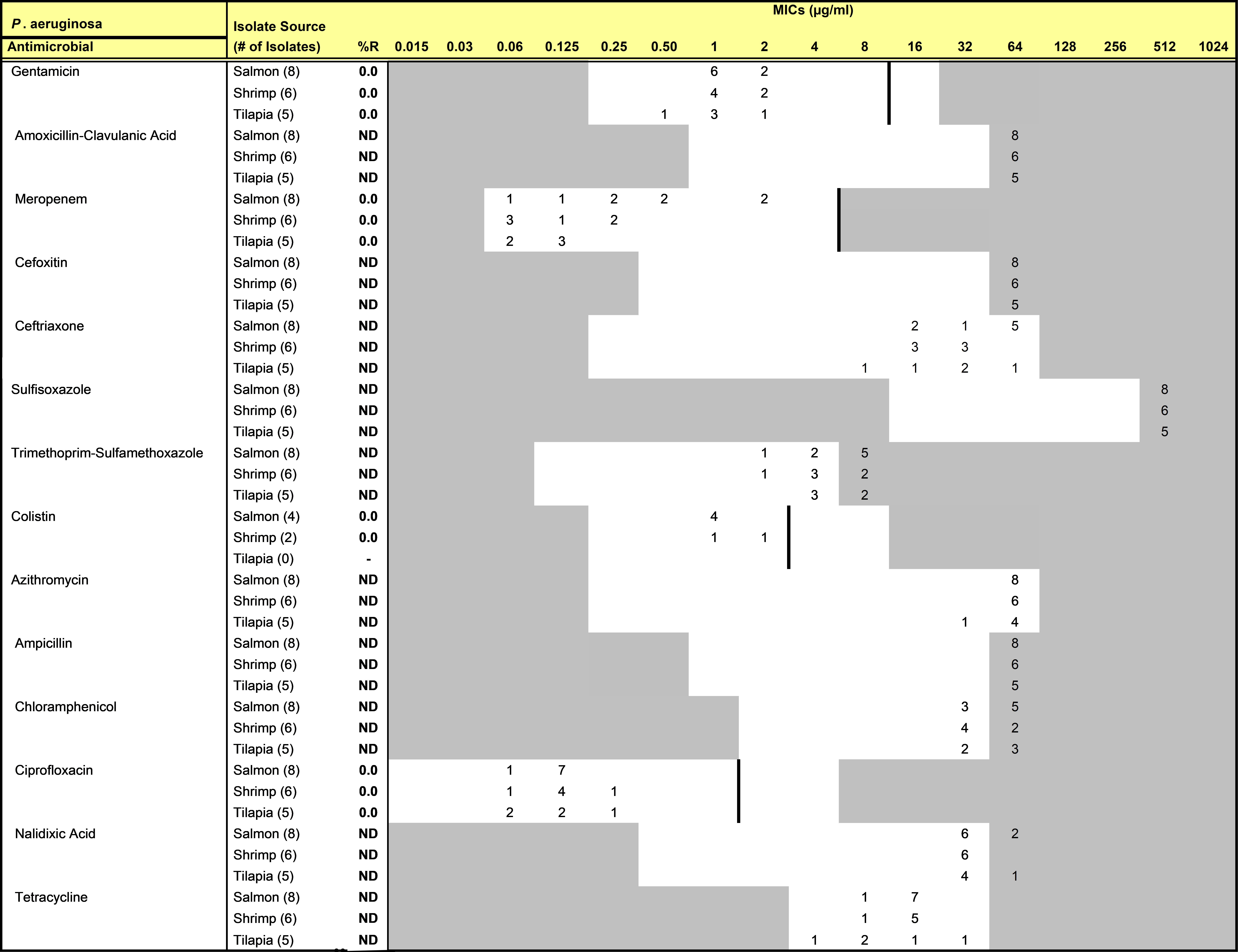

Supplement: Supplementary file 2 [file Image_2.JPEG]

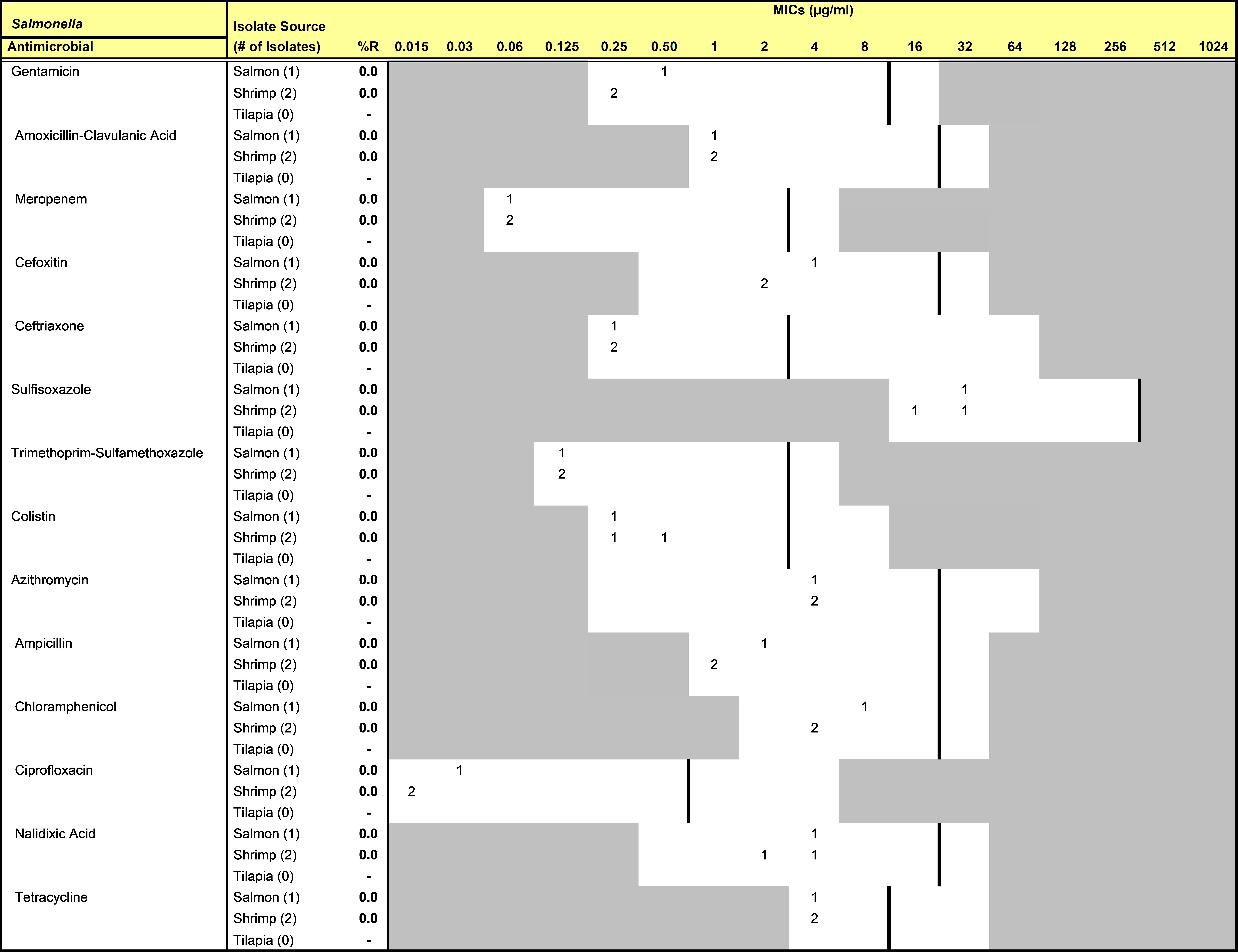

Supplement: Supplementary file 3 [file Image_3.JPEG]

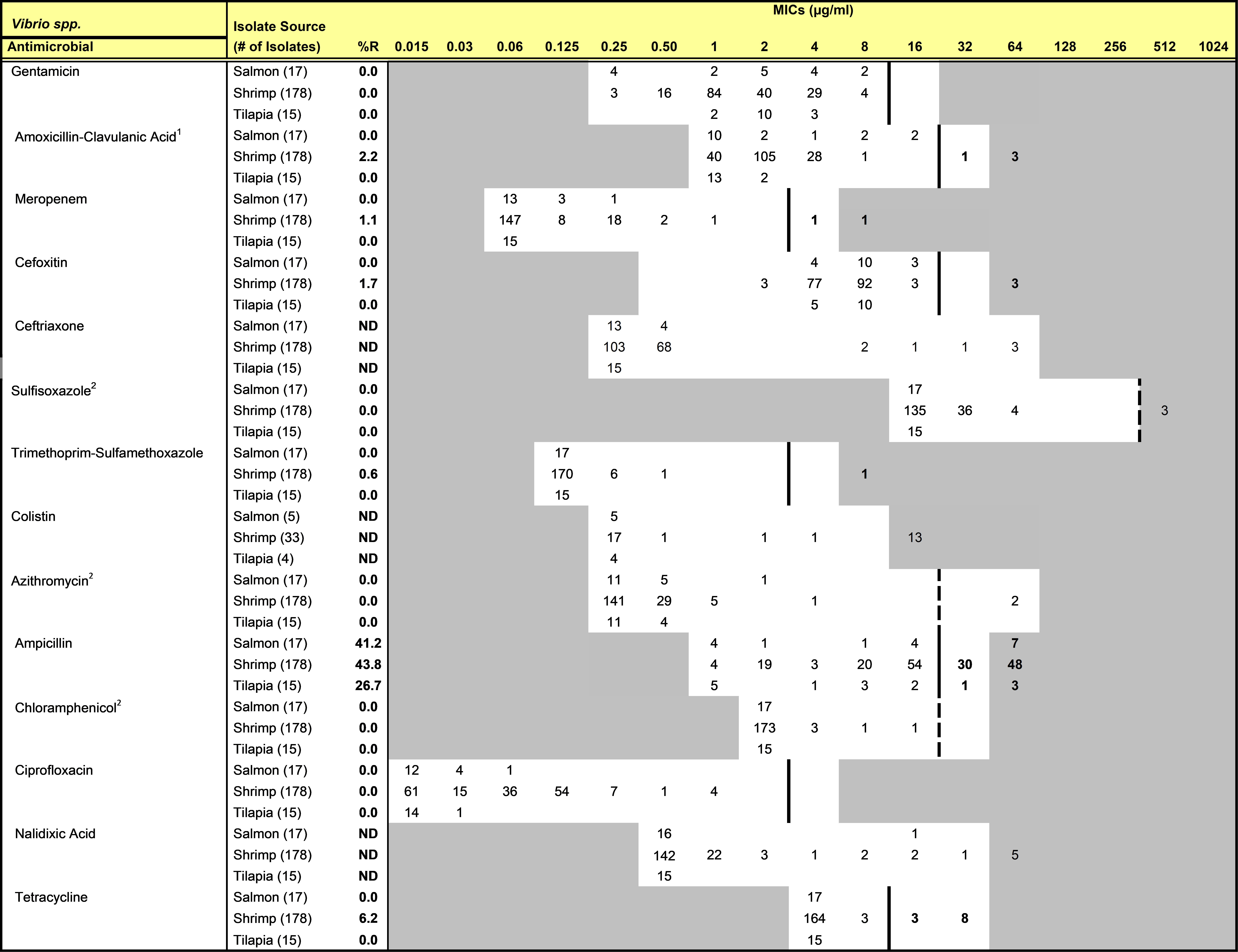

Supplement: Supplementary file 4 [file Image_4.JPEG]

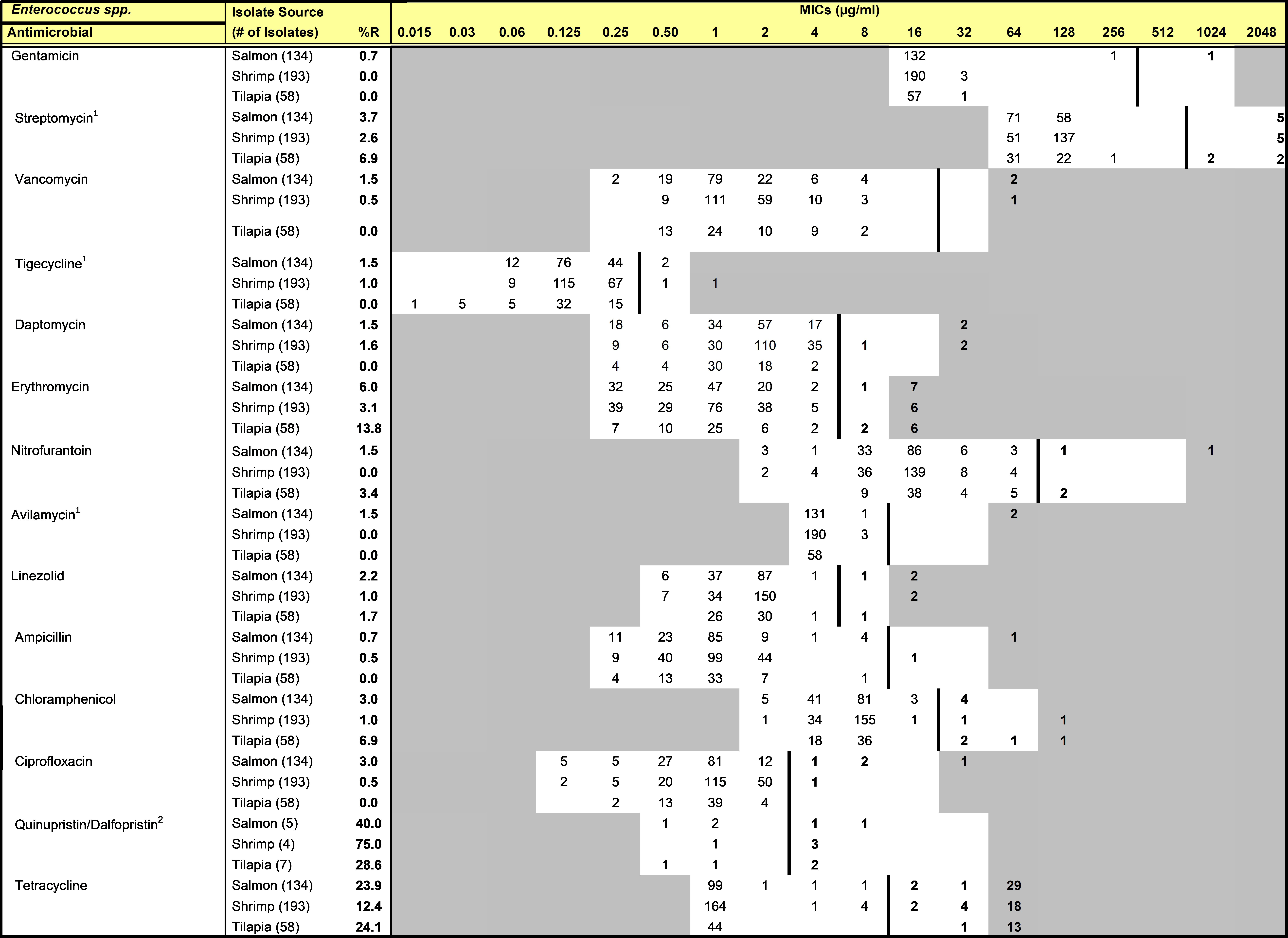

Supplement: Supplementary file 5 [file Image_5.JPEG]

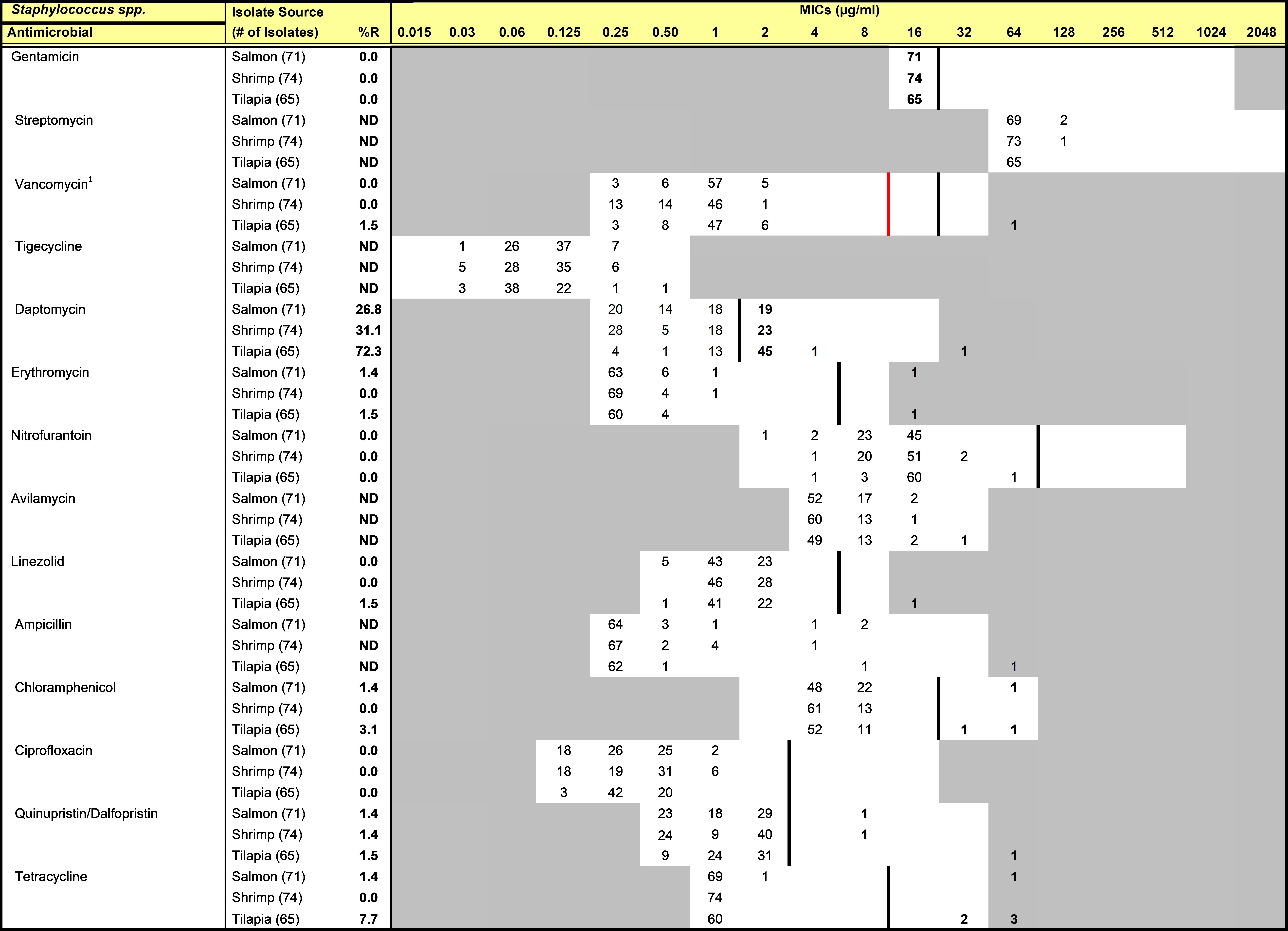

Supplement: Supplementary file 6 [file Image_6.JPEG]

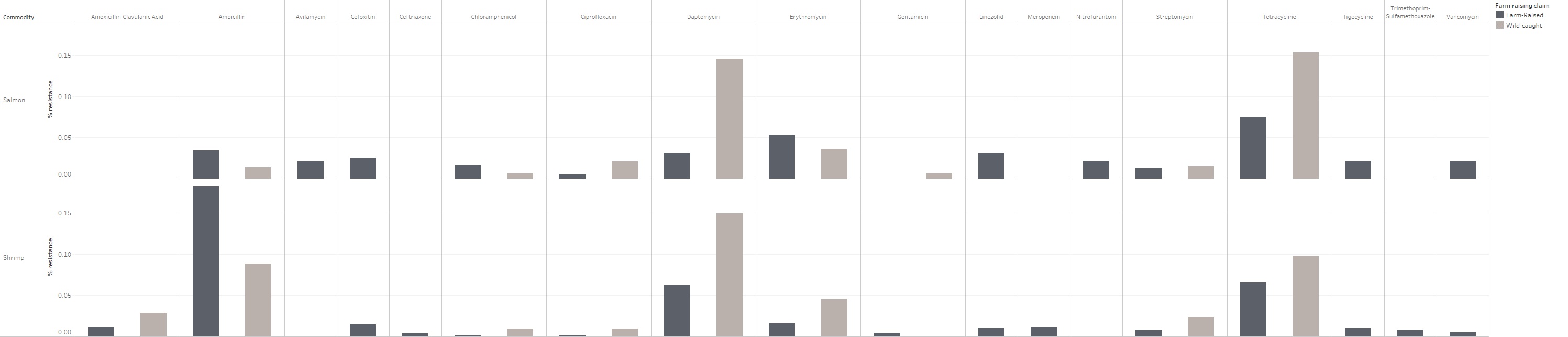

Supplement: Supplementary file 7 [file Image_7.JPEG]
