# Supplementary material for: Prevalence of Antimicrobial Resistance in Select Bacteria From Retail Seafood—United States, 2019
Source: Front Microbiol. 2022 Jun 23;13:928509. doi: 10.3389/fmicb.2022.928509 (PMC9262255; doi:10.3389/fmicb.2022.928509)
Supplement: Supplementary file 9 [file Data_Sheet_1.docx]

**Supporting Information**

**Supplemental Figure 1.** Distribution of MICs among *Aeromonas* spp. isolates from seafood.

**Supplemental Figure 2.** Distribution of MICs among *P. aeruginosa* isolates from seafood.

**Supplemental Figure 3.** Distribution of MICs among *Salmonella* isolates from salmon and shrimp.

**Supplemental Figure 4.** Distribution of MICs among *Vibrio* spp. isolates from seafood. ^1^Breakpoint applies to non-cholerae *Vibrio*; ^2^Breakpoint applies to *V. cholerae* only (dashed lines). If non-cholerae *Vibrio* have MICs above the breakpoint, they are not counted towards resistance calculations.

**Supplemental Figure 5.** Distribution of MICs among *Enterococcus* spp. isolates from seafood. ^1^NARMS established breakpoint; ^2^Testing results shown for confirmed *E. faecium* only.

**Supplemental Figure 6.** Distribution of MICs among *Staphylococcus* spp. isolates from seafood. ^1^The *S. aureus* resistance breakpoint is ≥ 16 µg/ml (red line) and the breakpoint for staphylococci other than *S. aureus* is ≥ 32 µg/ml (black lines).

**Supplemental Figure 7.** The proportion of all isolates (with interpretive criteria) resistant to antimicrobials by commodity and farm-raising claim.

**Supplemental Table S1.** Metadata for all 5596 isolates collected in the study
